# Pregnancy and Extra Uterine Conception

**Published:** 1859-06

**Authors:** George Higgins

**Affiliations:** Aurora, Illinois


ARTICLE II.

### PREGNANCY AND EXTRA UTERINE CONCEPTION.

BY GEORGE HIGGINS, M.D., OF AURORA, ILLINOIS.

On the 17th of March, 1859, I was called in haste to see Mrs. N. Orr. On my arrival, I found the patient suffering severe pain, just above and at the right of the symphysis pubis, with a sensation of bearing down.

Ascertaining the patient to be pregnant, made a vaginal examination. Nothing unusual presented; the os uteri firmly closed. Hoping to prevent an abortion, I gave a full dose of morphine, which relieved the pain, and the patient remained comfortable in bed, taking occasionally an opiate, moving the bowels in the meantime with oil.

All went on quietly, until the 19th, when the pain returned, with much more force than before, with a very unpleasant sensation in the lumbar region.

On making another examination, I detected an elastic tumor, high up in the vagina, in front of, and extending to, the uterus, which, at that time, was in the proper position, the pain rapidly increasing in severity.

As before, I gave a full anodyne, to quiet the pains, and waited the final termination, whatever it might be; as I left, telling the family to let me know, if there was any change in the symptoms.

On the 20th, at one o'clock A. M., I was called suddenly, the messenger stating the patient to be much worse. On arriving, I found the statement too correct.

Another examination revealed, to my great annoyance, the same elastic tumor, before mentioned, low down in the pelvis, crowding the uterus backwards and upwards, so that the ostincæ was out of reach. As the pains increased in severity, the tumor became tense, and seemed to occupy the whole cavity of the pelvis. I proposed counsel, as I thought the case would prove fatal. Dr. Howell was called. Soon after the Doctor entered the room, and while I was making an examination, the tumor gave way, followed by a large discharge of water, perhaps two quarts. Immediately after this rupture and discharge, the os was readily reached, and was not dilated in the least; satisfying me still better than before, that thet umor was external to and at the right of the uterus.

At this trying time, profuse hemorrhage set in, and not from the uterus, as might be supposed, but in front and at the right of that organ, from the direction in which the tumor was first discovered.

Dr. Howell satisfied himself that the flowing, which was constant, was not from the cavity of the uterus. After a short time, to our great relief, the mouth of the womb began to dilate. By means of the finger the dilation was more rapid, and in one hour's time, after the rupture of the tumor, or *sack*, two fœtus at five months were expelled, with the cords detached from the placentas, so that I was unable to remove them, the uterus closing down firmly.

As the patient at this time was very much prostrated, I thought best to preserve what little strength remained, as well as I could; so I gave a brandy sling, and a full dose of morphine, repeating the dose once in twenty minutes, until the pain was relieved, and reaction had taken place. Securing, by bandage and compression, a permanent contraction of the uterus, to prevent any hemorrhage that might occur from that direction, in one hour's time the patient was resting quietly. I left, to return again in two hours, ordering the nurse to let me know immediately, if any unfavorable symptoms should set in.

On my return, the nurse said the placenta had been expelled. As the patient remained quiet, I entertained a belief, or, rather, a hope, of there being but the one placenta; but, about two hours after, the husband came to me, saying, the pains had returned with great force. Sure enough, they had, and expelled the other placenta into the vagina. I removed it, and repeated the anodyne to control the pain; for, by this time, the patient was sighing, and almost gasping for breath. Pulse, one hundred and fifty per minute, and quite feeble.

By trifling manipulation, I succeeded in removing the tumor with membranes attached. Appeared much like a placenta, and weighing eight ounces; fetid, and somewhat decomposed. I would here state, the patient was as large as at full term, up to the rupture of the tumor. Now, where was the attachment, and what position did the tumor occupy, up to the morning of the 19th?

The previous history of the patient I have just received. In the summer of 1855, she became pregnant with her first child, a light-haired, blue-eyed boy. Since that time, her health has been declining, with soreness in the lower part of the abdomen, at the right of the symphysis pubis. Had noticed, at times, obstruction in the vagina, with extreme tenderness there.

In July last, aborted at three months, followed by a protracted illness of some four or five weeks.

Her last sickness was followed by no materially unfavorable symptoms. Says she feels better than at any time in the last three years. Is of scrofulous diathesis; tall, slender, with light hair and eyes; a native of Massachusetts, and thirty-two years of age.

